# Socio-occupational conditions and health of fishers exposed to the oil disaster-crime in Pernambuco, Brazil

**DOI:** 10.1590/1980-549720240050

**Published:** 2024-10-14

**Authors:** José Erivaldo Gonçalves, Ana Catarina Véras Medeiros Leite, Verônica Maria Cadena Lima, Wayner Vieira de Souza, Rita de Cássia Franco Rego, Mariana Olívia Santana dos Santos, Idê Gomes Dantas Gurgel

**Affiliations:** I Fundação Oswaldo Cruz, Aggeu Magalhães Institute, Department of Public Health – Recife (PE), Brazil.; II Universidade Federal da Bahia, Institute of Mathematics and Statistics, Department of Statistics – Salvador (BA), Brazil.; III Universidade Federal da Bahia, School of Medicine of Bahia – Salvador (BA), Brazil.; IV Universidade Federal do Rio Grande do Norte, Department of Public Health – Natal (RN), Brazil.

**Keywords:** Epidemiology, descriptive, Petroleum pollution, Public health, Environmental health

## Abstract

**Objective:**

To describe the sociodemographic, socio-occupational profile, and effects on the health of artisanal fishers from the state of Pernambuco, Brazil, affected by the oil disaster-crime in Brazil in 2019.

**Methods:**

This is a cross-sectional epidemiological study, carried out in 16 municipalities on the coast of Pernambuco, with a sample made up of 1,259 artisanal fishers. A questionnaire containing 14 blocks was used, including socioeconomic issues, exposure to oil, among others. A descriptive analysis was carried out with calculation of simple frequencies and percentages.

**Results:**

Of those interviewed, 95.1% considered fishing as their main occupation and 97% were carrying out this activity. Among fishers, the most common fishing spot was the mangrove, and wood fire was used in the work process by around 60% of the population. Regarding health issues, 34.4% reported a severe headache or migraine and 28.2% reported burning eyes, within one to three months after the oil spill.

**Conclusion:**

According to the results, working, health, and lifestyle conditions were impacted by the oil disaster-crime. Further research should be carried out to better understand the damage caused by exposure to oil and its effects on the health of fishers. Observing the profile of people who live in artisanal fishing territories in Pernambuco is paramount for public policies and government actions that promote safe and sustainable territories.

## INTRODUCTION

The National Policy on Holistic Health of Rural, Forest and Water Populations (*Política Nacional de Saúde Integral das Populações do Campo, da Floresta e das Águas* – PNSIPCFA) considers artisanal fishing communities as “peoples and communities with ways of life, production, and social reproduction predominantly related to aquatic environments”^1^. Artisanal fishers (AF) carry out small-scale fishing activities, either on land — such as shellfishing, in which they use wood fire in the fish processing stage, or on boats — fishing in rivers or open seas, for commercial purposes and, in addition to this condition, fishing assumes an affective and food consumption dimension for these populations^2^.

AF have a way of life linked to the healthy and sustainable productive and subsistence conditions of the environment — seas, rivers, estuaries, mangroves, among others^2^. These workers have relative work autonomy, threatened by development matrices that insert contexts of environmental injustice in the territories^3^. Environmental injustice, according to Acselrad^4^, is the unequal exposure of populations to risk arising from the capital accumulation model, introducing, in historically vulnerable territories, new processes that amplify this condition such as: real estate speculation, advancement of the petrochemical industry, and implementation of tourist complexes that affect social, economic, ecological, and health issues^5,6^.

The process of vulnerability occurs through the correlations of social, economic, and political forces in the territories, causing direct impacts on decision-making in health management, the formation of territorial policies and actions aimed at prevention and recovery in cases such as the disaster-crime of the oil spill that hit the Brazilian coast in 2019, especially the Northeast region^6,7^. This disaster-crime, considered a socio-environmental tragedy and one of the largest to occur in the South Atlantic and the largest on the Brazilian coast, caused environmental, socioeconomic, and health impacts, reflected in exposure to oil, food insecurity, economic vulnerability, and environmental contamination — mainly for artisanal fishing communities whose subsistence depends directly on fishing biodiversity^5,6^.

Regarding human health, hundreds of people were directly exposed to oil during beach cleaning activities, underestimating the effects on health^5^. Exposure to oil can have effects on mental and physical health, with genotoxic and immunotoxic repercussions and endocrine toxicity, which can manifest years after exposure, with women, older adults, and children being the most susceptible^8^.

Despite the cultural and productive importance of these populations, records of artisanal fishing workers are out of date, and existing data do not follow a standard of organization and standardization, making it difficult to obtain reliable information^9^. Associated with the lack of information on the profile of this population, there was a failure to monitor exposure and cases of poisoning during the oil disaster-crime, which led to difficulties in decision-making to tackle the disaster and implement social and health actions for affected communities^10^. The lack of information also caused delays in activating contingency plans and negligence in some scenarios^6^.

Knowledge of the general and unique aspects of these communities is essential to support social and health actions and policies that can be efficiently implemented in the territories. Thus, in this article we aim to describe and analyze the socio-occupational characteristics and some health effects of fishers exposed to the 2019 oil disaster-crime in the state of Pernambuco.

## METHODS

This is a cross-sectional epidemiological study carried out in the Northeast region of Brazil with data collected in 16 municipalities on the coast of Pernambuco affected by the oil disaster-crime in 2019, namely: Cabo de Santo Agostinho, Ipojuca, Rio Formoso, Sirinhaém, Tamandaré, Barreiros, São José da Coroa Grande, Goiana, Igarassu, Itapissuma, Itamaracá, Recife, Jaboatão dos Guararapes, Paulista, Olinda, Abreu, and Lima.

A validated questionnaire containing 13 blocks of questions addressing variables of exposure to oil and characterization of the social, environmental, economic, and health conditions of the population interviewed was used as a research instrument^11^. Another block with variables related to mental health was added to the original questionnaire, making a total of 14 blocks and 361 questions. To apply the questionnaire, the offline HCMaps application was used on electronic tablets , and each questionnaire took an average of 60 minutes to complete^12^. Data collection took place between September 2021 and August 2022.

The study population consisted of 12,472 fishers, registered in the 27 fishers’ colonies and/or associations on the coast of Pernambuco^13^. The inclusion criteria for participants were: individuals over 18 years of age and artisanal fishing workers who carried out the activity during the period of the oil disaster-crime. As an exclusion criterion, retired workers and/or those who were away from work during the oil disaster-crime were dismissed.

To define the sample, the coast of Pernambuco was stratified into three segments (North, Metropolitan, and South), adopting a study effect of 1.5 with a prevalence of 40% for health effects^14^, error of 3.5%, and confidence of 95%, resulting in a minimum sample size of 1,065 individuals. A 25% possibility of losses was added to the sample value, resulting in a sample of 1,331 people. Due to the number of registered people being approximately 1/3 of the total population, a proportional distribution was carried out for each coast and subsequently by the associations/colonies of each municipality. After completing the fieldwork, the research inclusion and exclusion criteria were adapted, obtaining a final sample of 1,259 people, exceeding the minimum sample size expected by approximately 18%.

For this study, blocks related to sociodemographic, health, and work conditions were considered, using the following variables: sex, age group, race/skin color, education, main occupation, work in fishing/shellfishing, work other than fishing, fishing spots, type of stove used for work, interruption of activity due to the oil spill, damage caused to usual fishing areas, location of oil while fishing, and main frequencies of symptoms presented in the period of one to three months after the start of the oil spill (nasal congestion, itchy or runny nose, severe headache or migraine, watery or itchy eyes, burning eyes). For data analysis, the absolute and relative percentage frequencies of the selected variables were calculated using the R v. 4.2.1 computational platform — available free of charge on cran.r-project.org.

## RESULTS

Among the 1,259 individuals participating in the study, 852 (67.7%) were women, 616 (48.9%) aged between 45 and 59 years, and 1,068 (84.8%) people declared themselves to be brown or Black; in relation to education, 71 (5.6%) were illiterate and 699 (55.6%) had some elementary school (Table 1).

**Table 1:** Distribution of sociodemographic variables of artisanal fishers in coastal municipalities of Pernambuco, 2024.

Variables	Metropolitan coast	North coast	South coast	Total
	(n=435)	(n=466)	(n=358)	(n=1,259)
	n (%)	n (%)	n (%)	n (%)
Sex				
Women	288 (66.2)	350 (75.1)	214 (59.8)	852 (67.7)
Men	147 (33.8)	116 (24.9)	144 (40.2)	407 (32.3)
Age group				
20–24	19 (4.4)	5 (1)	9 (2.5)	33 (2.6)
25–44	165 (37.9)	183 (39.3)	147 (41.1)	495 (39.3)
45–59	199 (45.8)	244 (52.4)	173 (48.3)	616 (48.9)
60+	52 (11.9)	34 (7.3)	29 (8.1)	115 (9.2)
Race/skin color				
Black/brown	359 (82.5)	407 (87.4)	302 (84.4)	1068 (84.8)
White	64 (14.7)	51 (10.9)	49 (13.7)	164 (13)
Indigenous	5 (1.1)	2 (0.4)	–	7 (0.6)
Others/Na*	7 (1.7)	6 (1.3)	7 (1.9)	20 (1.6)
Level of education				
Illiterate	25 (5.7)	29 (6.2)	17 (4.8)	71 (5.6)
Some elementary school	258 (59.3)	254 (54.5)	187 (52.2)	699 (55.5)
Elementary school	35 (8)	19 (4.1)	43 (12.0)	97 (7.7)
Some high school	30 (6.9)	36 (7.7)	27 (7.5)	93 (7.4)
High School	82 (18.9)	127 (27.3)	75 (20.9)	284 (22.6)
Some college/College degree	5 (1.2)	1 (0.2)	7 (2.0)	13 (1.0)

Source: Authors, 2024.

*Did not know how to answer it.

Artisanal fishing was considered the main occupation by 1,197 (95.1%) interviewees, and 1,208 (95.9%) were working in the fishing production process. Among those who carried out activities other than fishing, there were 62 (26.2%) individuals. Regarding the artisanal fishing work environment, 644 (51.2%) had the mangrove as their workplace. In turn, the wood-burning stove for work is used by 756 (60%) of them (Table 2).

**Table 2:** Working conditions of artisanal fishers in coastal municipalities of Pernambuco affected by the oil disaster-crime, 2024.

Variables	n	%
Is fishing your main occupation?		
Yes	1197	95.0
No	62	5.0
Do you currently work in fishing?		
Yes	1161	92.0
No	36	8.0
What are the fishing spots?		
Sand		
Yes	412	32.7
No	847	67.3
Seaside/open sea		
Yes	562	44.7
No	697	55.3
Mangrove		
Yes	644	51.2
No	615	48.8
What type of stove do you use for work?		
Bottled gas		
Yes	286	22.7
No	973	77.3
Wood-burning		
Yes	756	60.0
No	503	40.0

Source: Authors, 2024.

Regarding the fishing activity and the oil disaster-crime, 1,094 (86.9%) interviewees interrupted their work activity due to the oil spill. As for damage to regular fishing areas, 1,205 (95.7%) respondents reported damage caused by the disaster and 873 (69.3%) found oil while fishing (Table 3).

**Table 3:** Working conditions of artisanal fishers in coastal municipalities of Pernambuco affected by the oil disaster-crime, 2024.

Variables	n	%
Did you stop your activity because of the oil spill?		
Yes	1094	86.9
No	165	13.1
Did the disaster-crime cause damage to the usual fishing areas?		
Yes	1205	95.7
No	54	4.3
Did you find oil while fishing?		
Yes	873	69.3
No	386	30.7
Within one to three months of the oil spill, did you experience:		
Nasal congestion, itchy or runny nose?		
Yes	336	26.7
No	923	73.3
Severe headache or migraine?		
Yes	433	34.4
No	826	65.6
Watery or itchy eyes?		
Yes	303	24.1
No	956	75.9
Burning eyes?		
Yes	355	28.2
No	904	71.8

Source: Authors, 2024.

Among the symptoms self-reported by the population after exposure, during the period of one to three months after the oil disaster-crime, the neurological symptoms headache or migraine were two of the most frequent, accounting for 433 (34%) individuals, followed by the ocular system symptom burning eyes, for 355 (28.2%) people, and by nasal congestion, itchy or runny nose, symptoms of the respiratory system, for 336 (26.7%) individuals (Table 3).

## DISCUSSION

The majority of participants in this study are women and Black people with education level up to some elementary school, aged between 45 and 59 years, who consider fishing as their main occupation and are currently working. The mangrove was the environment most frequently mentioned as a fishing spot, and wood fire used during the work process was reported by more than half of the participants. We verified that most of the interviewees stopped fishing during the event and that the disaster caused damage to fishing areas. As for the oil exposure, a large percentage encountered oil while working, and about 1/3 of respondents reported symptoms related to the nervous, ocular, and respiratory systems.

In the sample, 67.7% of respondents were women, a statistic similar to recent data from the Brazilian Ministry of Fisheries and Aquaculture (2023)^15^, which released information on the artisanal fishing population, showing a greater number of fisherwomen compared to fishermen in Pernambuco^15^. Pena et al.^16^ identified that most artisanal fishing workers work in shellfishing, in mangroves and on the sand, and this is a predominantly female work. Although women play a prominent role in fishing, they are often made invisible^17,18^.

The proportion of Black people interviewed was similar to that found in the study by Mesquita and Quinamo^19^, reinforcing the historicity and Black representation in the history of artisanal fishing in Brazil^17,18,20^. There is a concentration of people between 45 and 59 years old, which creates a generational issue in the work and maintenance of artisanal fishing^16^.

The context of socio-environmental injustice and racism is largely responsible for the lack of or difficulty in accessing formal education in artisanal fishing territories, which has a negative impact on health and the construction of a dignified and happy life^21^. Alencar and Maia^22^ show in their study that around 75% of Brazilian fishers did not complete elementary school. In the Northeast, around 69.4% of fishers have some elementary school^19^, a proportion similar to that found in the present study. There is a need for educational strategies that value popular and scientific knowledge together, so that the way of life of these communities and the work process of these fishers can be understood and integrated — thus valuing artisanal fishing, the mobilization of traditional knowledge, and the guarantee of the right to decent work, health, and life^16,23,24^.

The government structure of artisanal fishing in the country presents conditions that often delegitimize the representative and autonomous exercise of fishers^25^. The relationship of these individuals with the territory and work is traced based on worldviews and cultural, social, and identity constructs that go beyond extractive and purely commercial logic^16,20^. This means that any change or alteration that has an impact on the fishing work process can create scenarios of food insecurity — as part of the fish is for personal consumption —, socioeconomic insecurity, changes in the traditional way of life, and harm to physical and mental health^10,26^.

The mangrove, where more than half of the participants in this study work, was directly affected by the oil spill in 2019. This ecosystem is considered a natural nursery and, therefore, very sensitive to environmental changes, which can cause damage to the genetic structure of species that live or reproduce in this habitat, triggering cascading effects of micro- and macroecological proportions^27,28^.

In addition to the repercussions on the environment, the effects on human health due to exposure to oil are diverse, affecting physical and mental health, and can be acute or chronic in the short, medium, or long term^8,29^. D’Andrea and Reddy^30^ demonstrated the development of changes or worsening of hematological, hepatic, pulmonary, and cardiac functions in people who worked in the cleanup during the oil spill on the Deepwater Horizon platform, even after seven years of exposure^30^.

There is also evidence of the development of headaches and ocular and respiratory symptoms even after 12 months after initial exposure to oil, recording a decrease in respiratory symptoms in relation to the first 30 days^31^. In terms of age, Jung et al.^32^ showed that children who lived near the Hebei Spirit oil spill disaster in South Korea had a higher prevalence of respiratory symptoms than those who lived far from the oil spill area^32^.

Among the oil components, 16 aromatic hydrocarbons (acenaphthene, acenaphthylene, anthracene, benzo[a]anthracene, benzo[a]pyrene, benzo[b]fluoranthene, benzo[ghi]perylene, benzo[k]fluoranthene, chrysene, dibenzo[a,h]anthracene, fluoranthene, fluorene, indeno [1,2,3-cd]pyrene, phenanthrene, pyrene, and naphthalene) are under priority monitoring by the United States Environmental Protection Agency — USEPA^33^. Benzene is considered a carcinogenic substance for which there is no safe exposure threshold, classified as a chemical that is highly harmful to human health^34^. The compound enters the body primarily through the respiratory tract, causing acute symptoms — such as headache, nausea, tachycardia, difficulty breathing, and loss of consciousness — and chronic symptoms — such as immune deficiency, dysregulation of the hematological system with a propensity for cancer, among other effects^35^.

Smoke from the use of wood-burning stoves during seafood processing is another factor of chemical exposure resulting from the work process of this population and which has health effects^16^. The smoke released during wood burning can be responsible for worsening respiratory diseases and increasing the risk of developing various types of cancer in the gastrointestinal tract^36,37^, causing greater harm to health when associated with the effects of intoxicants such as pesticides or oil compounds.

The intervention of large-scale projects in the territories, such as the implementation of petrochemical and automobile complexes and the occurrence of natural or technological disasters, such as the one that occurred in 2019, lead to the consequent contamination of the coastal ecosystem, increasing the risks inherent in work processes and introducing other harms to the environment, health, life, and social reproduction in these communities^10,23,24,26^.

In this study, we could observe vulnerabilities historically intertwined in the social reproduction of these communities in Brazil. There is a profile of low levels of education, predominantly Black people, who depend on artisanal fishing work and survive on their relationship with the environment and Anthropocene interventions. The interruption of work and the damage caused to the fishing environment can be seen as a possible factor impacting the living conditions and food sovereignty of these people. Freitas et al.^10^ emphasize the impacts of the oil spill on these communities.

In relation to health, the presence of symptoms suggests the need for further investigation into the health and illness process of these communities, and actions could be implemented to monitor health risks and the people exposed during the oil spill in order to detect early the damage caused and the effects on the health of these communities^8^.

The limitations of this study include the fact that it is a cross-sectional study that considers exposure and effect at the same point in time, not allowing long-term monitoring of these individuals and possible memory biases. Our results contribute to identifying the exposure process and the frequency of health complaints reported by fishers, requiring further studies to delve deeper into this topic and enable a greater understanding between exposure to oil and the health of these populations.

Observing the profile of people living in artisanal fishing territories in Pernambuco is paramount for thinking about strategies for implementing public policies and government actions to combat socio-environmental injustices and environmental racism, promoting healthy and sustainable territories that live in interdependence with the environment.
