# Erratum

**DOI:** 10.1590/1980-549720240050erratum

**Published:** 2024-11-22

In the manuscript "*Socio-occupational conditions and health of fishers exposed to the oil disaster-crime in Pernambuco, Brazil*", DOI: https://doi.org/10.1590/1980-549720240050, published in the Rev Bras Epidemiol. 2024; 27: e240050

**Where it reads:**

Ana Catarina Véras Medeiros Leite

**It should read:**

Ana Catarina Leite Veras Medeiros

**Where it reads:**

HOW TO CITE THIS ARTICLE: Gonçalves JE, Leite ACVM, Lima VMC, Souza WV, Rego RCF, Santos MOS, et al. Socio-occupational conditions and health of fishers exposed to the oil disaster-crime in Pernambuco, Brazil. Rev Bras Epidemiol. 2024; 27: e240050. https://doi.org/10.1590/1980-549720240050

**It should read:**

HOW TO CITE THIS ARTICLE: Gonçalves JE, Medeiros ACLV, Lima VMC, Souza WV, Rego RCF, Santos MOS, et al. Socio-occupational conditions and health of fishers exposed to the oil disaster-crime in Pernambuco, Brazil. Rev Bras Epidemiol. 2024; 27: e240050. https://doi.org/10.1590/1980-549720240050

**Where it reads:**

AUTHORS’ CONTRIBUTIONS: Gonçalves, J.E.: Formal analysis, Conceptualization, Data curation, Writing – original draft, Writing – review & editing, Investigation, Methodology, Funding acquisition, Validation. Leite, A.C.V.M.: Data curation, Writing – review & editing, Investigation, Methodology, Validation. Lima, V.M.C.: Data curation, Writing – review & editing, Methodology, Supervision, Validation, Visualization. Souza, W.V.: Data curation, Writing – review & editing, Methodology, Supervision, Validation, Visualization. Rego, R.C.F.: Writing – review & editing, Methodology, Supervision, Validation, Visualization. Santos, M.O.S.: Project administration, Conceptualization, Writing – review & editing, Investigation, Methodology, Funding acquisition, Supervision, Validation, Visualization. Gurgel, I.G.D.: Project administration, Conceptualization, Writing – review & editing, Investigation, Methodology, Funding acquisition, Supervision, Validation, Visualization.

**It should read:**

AUTHORS’ CONTRIBUTIONS: Gonçalves, J.E.: Formal analysis, Conceptualization, Data curation, Writing – original draft, Writing – review & editing, Investigation, Methodology, Funding acquisition, Validation. Medeiros, A.C.L.V.: Data curation, Writing – review & editing, Investigation, Methodology, Validation. Lima, V.M.C.: Data curation, Writing – review & editing, Methodology, Supervision, Validation, Visualization. Souza, W.V.: Data curation, Writing – review & editing, Methodology, Supervision, Validation, Visualization. Rego, R.C.F.: Writing – review & editing, Methodology, Supervision, Validation, Visualization. Santos, M.O.S.: Project administration, Conceptualization, Writing – review & editing, Investigation, Methodology, Funding acquisition, Supervision, Validation, Visualization. Gurgel, I.G.D.: Project administration, Conceptualization, Writing – review & editing, Investigation, Methodology, Funding acquisition, Supervision, Validation, Visualization.

