# Supplementary material for: Socio-occupational conditions and health of fishers exposed to the oil disaster-crime in Pernambuco, Brazil
Source: Rev Bras Epidemiol. 2024 Oct 14;27:e240050. doi: 10.1590/1980-549720240050 (PMC11481045; doi:10.1590/1980-549720240050)
Supplement: Translation

# Translation: Condições socio-ocupacionais e saúde de pescadores expostos ao desastre-crime do petróleo em Pernambuco

**DOI:** 10.1590/1980-549720240050.2

**Authors:** José Erivaldo Gonçalves, Ana Catarina Véras Medeiros Leite, Verônica Maria Cadena Lima, Wayner Vieira de Souza, Rita de Cássia Franco Rego, Mariana Olívia Santana dos Santos, Idê Gomes Dantas Gurgel

**Affiliations:** I Fundação Oswaldo Cruz, Instituto Aggeu Magalhães, Departamento de Saúde Coletiva – Recife (PE), Brasil.; II Universidade Federal da Bahia, Instituto de Matemática e Estatística, Departamento de Estatística – Salvador (BA), Brasil.; III Universidade Federal da Bahia, Faculdade de Medicina da Bahia – Salvador (BA), Brasil.; IV Universidade Federal do Rio Grande do Norte, Departamento de Saúde Coletiva – Natal (RN), Brasil.

**Keywords:** Epidemiologia descritiva, Derramamentos de petróleo, Saúde pública, Saúde ambiental

## Abstract

**Objetivo:**

Descrever os perfis sociodemográfico e socio-ocupacional e efeitos na saúde dos pescadores artesanais de Pernambuco afetados pelo desastre-crime do petróleo no Brasil em 2019.

**Métodos:**

Estudo epidemiológico transversal, realizado em 16 municípios do litoral pernambucano, com amostra composta por 1.259 pescadores artesanais. Foi utilizado um questionário contendo 14 blocos, incluindo questões socioeconômicas, de exposição ao petróleo, entre outras. Foi realizada uma análise descritiva com cálculo de frequências simples e percentual.

**Resultados:**

No total, 95,1% das pessoas consideram a pesca como seu principal trabalho e 97% estavam exercendo essa atividade. Entre os pescadores, o local de pesca mais comum foi o mangue, e o fogo à lenha foi utilizado no processo de trabalho por cerca de 60% da população. Em relação a problemas de saúde, 34,4% relataram dor de cabeça forte ou enxaqueca e 28,2%, ardência nos olhos, no período de 1 a 3 meses após o derramamento de petróleo.

**Conclusão:**

As condições de trabalho, de saúde e de estilo de vida foram impactadas pelo desastre-crime do petróleo. Outras pesquisas deverão ser desenvolvidas para melhor compreender os danos da exposição ao petróleo e seus efeitos na saúde dos pescadores. Observar o perfil das pessoas que vivem nos territórios da pesca artesanal em Pernambuco é fundamental para políticas públicas e ações governamentais que promovam territórios saudáveis e sustentáveis.

## INTRODUÇÃO

A Política Nacional de Saúde Integral das Populações do Campo, da Floresta e das Águas (PNSIPCFA) considera as comunidades da pesca artesanal como “povos e comunidades com modos de vida, produção e reprodução social relacionados predominantemente com os ambientes aquáticos”^1^. Os pescadores artesanais (PA) realizam atividades de pesca em pequena escala, desembarcados — como a pesca de mariscos, em que utilizam o fogo à lenha na etapa de processamento do pescado, ou em embarcações — pesca em rio ou mar aberto, para fins de comercialização e, para além desta condição, a pesca assume uma dimensão afetiva e de consumo alimentar dessas populações^2^.

Os PA possuem um modo de vida atrelado às condições produtivas saudáveis e sustentáveis e de subsistência do ambiente — mares, rios, estuários, manguezais, dentre outros^2^. Esses trabalhadores possuem uma autonomia de trabalho relativa, ameaçada por matrizes de desenvolvimento que inserem contextos de injustiça ambiental nos territórios^3^. A injustiça ambiental, de acordo com Acselrad^4^, é a exposição desigual de populações ao risco proveniente do modelo de acumulação de capital, introduzindo, nos territórios historicamente vulnerabilizados, novos processos que ampliam essa condição, tais como: especulação imobiliária, avanço da indústria petroquímica e implantação de complexos turísticos que incidem em questões sociais, econômicas, ecológicas e de saúde^5,6^.

O processo de vulnerabilização acontece mediante as correlações de forças sociais, econômicas e políticas nos territórios, causando impactos diretos nas tomadas de decisão da gestão em saúde, formação de políticas territoriais e das ações direcionadas para a prevenção e recuperação em casos como o desastre-crime do derramamento de petróleo que atingiu o litoral brasileiro em 2019, sobretudo a região Nordeste^6,7^. Esse desastre-crime, considerado uma tragédia socioambiental e um dos maiores ocorridos no atlântico Sul e o maior da costa brasileira, causou impactos ambientais, socioeconômicos e na saúde, refletidos na exposição ao petróleo, insegurança alimentar, vulnerabilidade econômica e contaminação do ambiente — principalmente para as comunidades da pesca artesanal que vivem diretamente da biodiversidade pesqueira^5,6^.

Em relação à saúde humana, centenas de pessoas foram expostas diretamente ao petróleo durante a atuação na limpeza das praias, subdimensionando os efeitos na saúde^5^. A exposição ao petróleo pode gerar efeitos na saúde mental e física, com repercussões genotóxicas e imunotóxicas e toxicidade endócrina, as quais podem se manifestar anos após a exposição, sendo mulheres, idosos e crianças os mais suscetíveis^8^.

Apesar da importância cultural e produtiva dessas populações, os registros dos trabalhadores da pesca artesanal estão desatualizados, e os dados existentes não seguem um padrão de organização e uniformização, dificultando a obtenção de informações fidedignas^9^. Associado à ausência de informações sobre o perfil dessa população, houve uma falha no monitoramento da exposição e dos casos de intoxicação durante o desastre-crime do petróleo, o que implicou em dificuldades nas tomadas de decisão para o enfrentamento do desastre e a implementação de ações sociais e em saúde para comunidades atingidas^10^. A ausência de informações também provocou morosidade na ativação de planos de contingência e negligência em alguns cenários^6^.

O conhecimento dos aspectos gerais e singulares dessas comunidades é fundamental para o subsídio de ações e políticas sociais e em saúde que podem ser implementadas de forma eficiente nos territórios. Assim, este artigo pretende descrever e analisar as características socio-ocupacionais e alguns efeitos na saúde de pescadores expostos ao desastre-crime do petróleo em 2019 no estado de Pernambuco.

## MÉTODOS

Estudo epidemiológico de corte transversal, realizado na região Nordeste do Brasil com dados coletados em 16 municípios do litoral de Pernambuco afetados pelo desastre-crime do petróleo em 2019, sendo eles: Cabo de Santo Agostinho, Ipojuca, Rio Formoso, Sirinhaém, Tamandaré, Barreiros, São José da Coroa Grande, Goiana, Igarassu, Itapissuma, Itamaracá, Recife, Jaboatão dos Guararapes, Paulista, Olinda, Abreu e Lima.

Foi utilizado, como instrumento de pesquisa, um questionário validado contendo 13 blocos de questões abordando variáveis de exposição ao petróleo e caracterização das condições sociais, ambientais, econômicas e de saúde da população entrevistada^11^. Foram acrescidas ao questionário original mais um bloco com variáveis relacionadas à saúde mental, constituindo no total 14 blocos e 361 perguntas. Para aplicação do questionário, foi utilizado o aplicativo *offline HCMaps* em *tablets* eletrônicos, e cada questionário durou em média 60 minutos de aplicação^12^. A coleta de dados ocorreu entre setembro de 2021 e agosto de 2022.

A população do estudo foi composta por 12.472 pescadores, registrados nas 27 colônias e/ou associações de pescadores(as) do litoral de Pernambuco^13^. Os critérios de inclusão dos participantes foram: indivíduos maiores de 18 anos e trabalhadores da pesca artesanal que exerciam a atividade durante o período do desastre-crime do petróleo. Como critério de exclusão, foram dispensados trabalhadores aposentados e/ou que estavam afastados do trabalho durante o desastre-crime do petróleo.

Para definição da amostra, o litoral pernambucano foi estratificado em três segmentos (Norte, Metropolitano e Sul), adotando um efeito de estudo de 1.5 com prevalência de 40% para efeitos na saúde^14^, erro de 3,5% e confiança de 95%, resultando em um tamanho de amostra mínimo de 1.065 indivíduos. Foram adicionadas ao valor amostral as possibilidades de perdas na ordem de 25%, resultando em uma amostra de 1.331 pessoas. Devido ao número de cadastrados ser aproximadamente 1/3 do total da população, foi realizada uma distribuição proporcional para cada litoral e em seguida pelas associações/colônias de cada município. Após a finalização do trabalho de campo, foi realizada a adequação aos critérios de inclusão e exclusão da pesquisa, obtendo-se uma amostra final de 1.259 pessoas, superando em aproximadamente 18% o tamanho mínimo de amostra previsto.

Para este estudo, foram considerados os blocos relacionados às condições sociodemográficas, de saúde e trabalho, utilizando-se as seguintes variáveis: sexo, faixa etária, raça/cor da pele, escolaridade, trabalho principal, trabalho na pesca/mariscagem, trabalho além da pesca, locais de pesca, tipo de fogão utilizado para o trabalho, interrupção da atividade devido ao derramamento de petróleo, danos ocasionados às áreas habituais de pesca, localização de petróleo enquanto pescava e principais frequências de sintomas apresentados no período de 1–3 meses após o início do derramamento de óleo/petróleo (nariz entupido, coceira ou corrimento nasal, forte dor de cabeça ou enxaqueca, olhos lacrimejantes ou com coceira, ardência nos olhos). Para a análise dos dados, foram calculadas as frequências absoluta e relativa percentual das variáveis selecionadas, através da plataforma computacional R v. 4.2.1 — disponível gratuitamente em *cran.r-project.org* .

## RESULTADOS

Entre os 1.259 indivíduos participantes do estudo, 852 (67,7%) eram do sexo feminino, 616 (48,9%) possuíam idades na faixa etária de 45-59 anos e 1.068 (84,8%) pessoas se autodeclararam pardas e pretas; em relação à escolaridade, 71 (5,6%) eram analfabetos e 699 (55,6%) possuíam o ensino fundamental incompleto (EFI) (Tabela 1).

**Tabela 1:** Distribuição das variáveis sociodemográficas de pescadores artesanais nos municípios do litoral de Pernambuco, 2024.

Variáveis	Litoral metropolitano	Litoral norte	Litoral sul	Total
	(n=435)	(n=466)	(n=358)	(n=1.259)
	n (%)	n (%)	n (%)	n (%)
Sexo				
Feminino	288 (66,2)	350 (75,1)	214 (59,8)	852 (67,7)
Masculino	147 (33,8)	116 (24,9)	144 (40,2)	407 (32,3)
Faixa etária				
20–24	19 (4,4)	5 (1)	9 (2,5)	33 (2,6)
25–44	165 (37,9)	183 (39,3)	147 (41,1)	495 (39,3)
45–59	199 (45,8)	244 (52,4)	173 (48,3)	616(48,9)
60+	52 (11,9)	34 (7,3)	29 (8,1)	115 (9,2)
Raça/cor de pele				
Preta/parda	359 (82,5)	407 (87,4)	302(84,4)	1068 (84,8)
Branca	64 (14,7)	51 (10,9)	49 (13,7)	164 (13)
Indígena	5 (1,1)	2 (0,4)	-	7 (0,6)
Outras/Nsr*	7 (1,7)	6 (1,3)	7 (1,9)	20 (1,6)
Escolaridade				
Analfabeto	25 (5,7)	29 (6,2)	17 (4,8)	71 (5,6)
Ensino fundamental incompleto	258 (59,3)	254 (54,5)	187 (52,2)	699 (55,5)
Ensino fundamental completo	35 (8)	19 (4,1)	43 (12,0)	97 (7,7)
Ensino médio incompleto	30 (6,9)	36 (7,7)	27 (7,5)	93 (7,4)
Ensino médio completo	82 (18,9)	127 (27,3)	75 (20,9)	284 (22,6)
Ensino superior incompleto/completo	5 (1,2)	1 (0,2)	7 (2,0)	13 (1,0)

Fonte: Autores, 2024.

*Não soube responder.

A pesca artesanal foi considerada como o principal trabalho por 1.197 (95,1%) entrevistados, e 1.208 (95,9%) estavam trabalhando no processo produtivo da pesca. Dentre os que exerciam outras atividades além da pesca, somam-se 62 (26,2%) indivíduos. Quanto ao ambiente de trabalho da pesca artesanal, 644 (51,2%) tinham o mangue como seu lugar de trabalho. Por sua vez, o fogão à lenha para o trabalho é utilizado por 756 (60%) deles (Tabela 2).

**Tabela 2:** Condições de trabalho de pescadores artesanais dos municípios do litoral de Pernambuco afetados pelo desastre-crime do petróleo, 2024.

Variáveis	n	%
A pesca é o principal trabalho?		
Sim	1197	95,0
Não	62	5,0
Atualmente trabalha na pesca?		
Sim	1161	92,0
Não	36	8,0
Quais os locais de pesca?		
Areia		
Sim	412	32,7
Não	847	67,3
Beira-mar/mar aberto		
Sim	562	44,7
Não	697	55,3
Mangue		
Sim	644	51,2
Não	615	48,8
Que tipo de fogão você utiliza para o trabalho?		
Gás de botijão		
Sim	286	22,7
Não	973	77,3
Lenha		
Sim	756	60,0
Não	503	40,0

Fonte: Autores, 2024.

Observando a atividade da pesca e o desastre-crime do petróleo, 1.094 (86,9%) entrevistados interromperam a atividade laboral por conta do derramamento de petróleo. No que diz respeito aos danos às áreas habituais da pesca, 1.205 (95,7%) entrevistados afirmaram danos ocasionados pelo desastre e 873 (69,3%) encontraram petróleo enquanto pescavam (Tabela 3).

**Tabela 3:** Condições de trabalho e saúde de pescadores artesanais dos municípios do litoral de Pernambuco afetados pelo desastre-crime do petróleo, 2024.

Variáveis	n	%
Interrompeu a atividade por causa do derramamento de petróleo?		
Sim	1094	86,9
Não	165	13,1
O desastre-crime ocasionou danos às áreas habituais de pesca?		
Sim	1205	95,7
Não	54	4,3
Encontrou petróleo enquanto pescava?		
Sim	873	69,3
Não	386	30,7
No período de 1–3 meses após o início do derramamento de petróleo, você apresentou:		
Nariz entupido, coceira ou corrimento nasal		
Sim	336	26,7
Não	923	73,3
Forte dor de cabeça ou enxaqueca		
Sim	433	34,4
Não	826	65,6
Olhos lacrimejantes ou com coceira		
Sim	303	24,1
Não	956	75,9
Ardência nos olhos		
Sim	355	28,2
Não	904	71,8

Fonte: Autores, 2024

Dentre os sintomas autorreferidos pela população após a exposição, durante o período de um a três meses após o desastre-crime do petróleo, os sintomas neurológicos dor de cabeça ou enxaqueca se apresentaram como dois dos mais frequentes, 433 (34%), seguidos do sintoma do sistema ocular ardência nos olhos para 355 (28,2%) e pelo nariz entupido, coceira ou corrimento nasal, sintomas do sistema respiratório, para 336 (26,7%) (Tabela 3).

## DISCUSSÃO

A maioria dos participantes deste estudo são mulheres e pessoas negras com um nível escolar até ensino fundamental incompleto, com perfil etário entre 45 e 59 anos, que considera a pesca como seu principal trabalho e estão em atividade. O mangue foi o ambiente mais frequentemente mencionado enquanto local de pesca, e o fogo a lenha utilizado durante o processo de trabalho foi relatado por mais da metade dos participantes. Verificou-se que a maioria dos entrevistados parou de pescar durante o evento e que o desastre causou danos aos locais de pesca. Em relação à exposição ao petróleo, uma grande porcentagem encontrou petróleo enquanto trabalhava, e cerca de 1/3 dos entrevistados relatou a presença de sintomas relacionados ao sistema nervoso, ocular e respiratório.

Na amostra, 67,7% de entrevistados eram do sexo feminino, estatística semelhante aos dados recentes do Ministério da Pesca e Aquicultura (2023)^15^, que divulgou informações sobre a população da pesca artesanal, evidenciando um número maior de mulheres pescadoras em relação aos homens em Pernambuco^15^. Pena et al.^16^ identificaram que a maior parte dos trabalhadores da pesca artesanal atuam na coleta de mariscos, nos mangues e na areia, sendo este um trabalho predominantemente feminino. Apesar de as mulheres terem destaque no trabalho da pesca, elas são muitas vezes invisibilizadas^17,18^.

A proporção de pessoas da cor/raça negra entrevistadas foi similar à encontrada no estudo de Mesquita e Quinamo^19^, reforçando a historicidade e a representação negra na história da pesca artesanal no Brasil^17,18,20^. Há uma concentração de pessoas entre 45 e 59 anos, que incide uma questão geracional no trabalho e na manutenção da pesca artesanal^16^.

O contexto de injustiça e racismo socioambiental é responsável, em grande medida, pela falta ou dificuldade do acesso à educação formal nos territórios da pesca artesanal, o que repercute de forma negativa na saúde e na construção de uma vida digna e feliz^21^. Alencar e Maia^22^ mostram em seu estudo que cerca de 75% dos pescadores brasileiros não concluíram o ensino fundamental. No Nordeste, cerca de 69,4% dos pescadores têm o ensino fundamental incompleto^19^, proporção similar ao encontrado no presente estudo. São necessárias estratégias de educação que valorizem o conhecimento popular e o científico articulados, de forma que se compreenda e se integre o modo de vida dessas comunidades e o processo de trabalho desses pescadores — valorizando, assim, a pesca artesanal, a mobilização de saberes tradicionais e a garantia do direito ao trabalho digno, à saúde e à vida^16,23,24^.

A estrutura governativa da pesca artesanal no país apresenta condições que, muitas vezes, deslegitimam o exercício representativo e autônomo dos pescadores^25^. A relação desses indivíduos com o território e o trabalho é traçada a partir de cosmovisões e constructos culturais, sociais e identitários que transpassam a lógica extrativista e puramente comercial^16,20^. Isso significa que qualquer mudança ou alteração que tenha impacto no processo de trabalho da pesca pode configurar cenários de insegurança alimentar — visto que parte do pescado é para consumo próprio —, insegurança socioeconômica, alteração no modo de vida tradicional e agravos à saúde física e mental^10,26^.

O mangue, lugar onde mais da metade dos participantes deste estudo trabalha, foi afetado diretamente pelo derramamento de petróleo em 2019. Este ecossistema é considerado um berçário natural e, por isso, muito sensível às alterações ambientais, que podem provocar danos na estrutura genética das espécies que vivem ou se reproduzem nesse habitat, desencadeando efeitos em cascata de proporções micro e macroecológicas^27,28^.

Além das repercussões no ambiente, os efeitos à saúde humana pela exposição ao petróleo são diversos, afetando a saúde física e mental, podendo apresentar-se de forma aguda ou crônica num espectro de tempo curto, médio ou longo^8,29^. Os autores D’Andrea e Reddy^30^ demonstraram o aparecimento de alterações ou piora de funções hematológicas, hepáticas, pulmonares e cardíacas em pessoas que trabalharam na limpeza durante o derramamento de petróleo na plataforma Deepwater Horizon, mesmo após sete anos da exposição^30^.

Há, também, evidências do aparecimento de dores de cabeça e sintomas oculares e respiratórios mesmo depois de 12 meses após a exposição inicial ao petróleo, registrando uma diminuição dos sintomas respiratórios em relação aos primeiros 30 dias^31^. Em recorte etário, Jung et al.^32^ apresentam que crianças que moravam nas proximidades de onde ocorreu o desastre *Hebei Spirit Oil Spill,* na Coreia do Sul, apresentavam uma maior prevalência de sintomas respiratórios do que aquelas que viviam longe da área de derramamento do petróleo^32^.

Entre os componentes do petróleo, 16 hidrocarbonetos aromáticos (acenafteno, acenaftileno, antraceno, benzo(a)antraceno, benzo(a)pireno, benzo(b) fluoranteno, benzo(ghi)perileno, benzo(K)fluoranteno, criseno, dibenzo(a,h)antraceno, fluoranteno, fluoreno, indeno [1,2,3-c,d] pireno, fenantreno, pireno e naftaleno) estão sob monitoramento prioritário pela *United States Environmental Protection Agency* — USEPA^33^. O benzeno é considerado uma substância cancerígena a qual não há um limiar seguro de exposição, classificado como um produto químico altamente nocivo para a saúde humana^34^. A entrada do composto no organismo acontece prioritariamente por via respiratória, provocando sintomas agudos, como cefaleia, náuseas, taquicardia, dificuldade respiratória, perda da consciência; e crônicos, por exemplo, deficiência imunológica, desregulação do sistema hematológico com propensão a câncer, entre outros efeitos^35^.

A fumaça do uso do fogão à lenha durante o processamento do marisco é outro fator de exposição química decorrente do processo de trabalho dessa população e que implica em efeitos na saúde^16^. A fumaça expelida durante a queima da madeira pode ser responsável pelo agravamento de doenças respiratórias e por aumentar o risco no desenvolvimento de diversos tipos de câncer do trato gastrointestinal^36,37^, gerando maiores prejuízos na saúde quando associada aos efeitos de intoxicantes como agrotóxicos ou compostos do petróleo.

A intervenção dos grandes empreendimentos nos territórios, como a implantação de complexos petroquímicos e automobilísticos e a ocorrência de desastres naturais ou tecnológicos, como o ocorrido em 2019, levam a consequente contaminação do ecossistema costeiro, potencializando os riscos inerentes aos processos de trabalho e introduzindo outros agravos ao ambiente, à saúde, à vida e à reprodução social nessas comunidades^10,23,24,26^.

É possível observar, neste estudo, vulnerabilidades historicamente imbricadas na reprodução social dessas comunidades no Brasil. Há um perfil de baixo nível de escolaridade, pessoas predominantemente negras, que dependem do trabalho da pesca artesanal e sobrevivem a partir da relação com o ambiente e de intervenções do antropoceno. A interrupção do trabalho e os danos causados ao ambiente de pesca podem ser percebidos como um possível fator de impacto nas condições de vida e na soberania alimentar dessas pessoas. Um estudo desenvolvido por Freitas et al.^10^ reforça impactos do derramamento do petróleo nessas comunidades.

Em relação à saúde, a presença dos sintomas sugere a necessidade de maiores investigações sobre a saúde e o processo de adoecimento dessas comunidades, podendo ser implementadas ações de monitoramento dos riscos à saúde e das pessoas expostas durante o derramamento de petróleo a fim de detectar precocemente os danos causados e os efeitos na saúde dessas comunidades^8^.

Apresenta-se como limitações ser um estudo de corte transversal que considera a exposição e o efeito em um mesmo momento no tempo, não possibilitando o acompanhamento a longo prazo desses indivíduos e possíveis vieses de memória. Os resultados descritos contribuem para identificar o processo de exposição e a frequência de queixas de saúde relatadas pelos pescadores, sendo necessário outros estudos que aprofundem essa temática e viabilizem uma maior compreensão entre a exposição ao petróleo e a saúde dessas populações.

Observar o perfil das pessoas que vivem nos territórios da pesca artesanal em Pernambuco é fundamental para pensar estratégias de implementação de políticas públicas e ações governamentais para combater as injustiças socioambientais e o racismo ambiental, promovendo territórios saudáveis e sustentáveis que vivem em interdependência com o ambiente.
