# Supplementary material for: Erratum
Source: Rev Bras Epidemiol. 2024 Nov 22;27:e240050erratum. doi: 10.1590/1980-549720240050erratum (PMC11654284; doi:10.1590/1980-549720240050erratum)
Supplement: Related texts

# Correction: Errata

**DOI:** 10.1590/1980-549720240050.2erratum

No artigo "*Condições socio-ocupacionais e saúde de pescadores expostos ao desastre-crime do petróleo em Pernambuco*", DOI: https://doi.org/10.1590/1980-549720240050.2, publicado no periódico Rev Bras Epidemiol. 2024; 27: e240050

**Onde se lia:**

Ana Catarina Véras Medeiros Leite

**Leia-se:**

Ana Catarina Leite Veras Medeiros

**Onde se lia:**

COMO CITAR ESSE ARTIGO: Gonçalves JE, Leite ACVM, Lima VMC, Souza WV, Rego RCF, Santos MOS, et al. Condições socio-ocupacionais e saúde de pescadores expostos ao desastre-crime do petróleo em Pernambuco. Rev Bras Epidemiol. 2024; 27: e240050. https://doi.org/10.1590/1980-549720240050.2

**Leia-se:**

COMO CITAR ESSE ARTIGO: Gonçalves JE, Medeiros ACLV, Lima VMC, Souza WV, Rego RCF, Santos MOS, et al. Condições socio-ocupacionais e saúde de pescadores expostos ao desastre-crime do petróleo em Pernambuco. Rev Bras Epidemiol. 2024; 27: e240050. https://doi.org/10.1590/1980-549720240050.2

**Onde se lia:**

CONTRIBUIÇÕES DOS AUTORES: Gonçalves, J.E.: Análise formal, Conceituação, Curadoria dos dados, Escrita – primeira redação, Escrita – revisão e edição, Investigação, Metodologia, Obtenção de financiamento, Validação. Leite, A.C.V.M.: Curadoria dos dados, Escrita – revisão e edição, Investigação, Metodologia, Validação. Lima, V.M.C.: Curadoria dos dados, Escrita – revisão e edição, Metodologia, Supervisão, Validação, Visualização. Souza, W.V.: Curadoria dos dados, Escrita – revisão e edição, Metodologia, Supervisão, Validação, Visualização. Rego, R.C.F.: Escrita – revisão e edição, Metodologia, Supervisão, Validação, Visualização. Santos, M.O.S.: Administração do projeto, Conceituação, Escrita – revisão e edição, Investigação, Metodologia, Obtenção de financiamento, Supervisão, Validação, Visualização. Gurgel, I.G.D.: Administração do projeto, Conceituação, Escrita – revisão e edição, Investigação, Metodologia, Obtenção de financiamento, Supervisão, Validação, Visualização.

**Leia-se:**

CONTRIBUIÇÕES DOS AUTORES: Gonçalves, J.E.: Análise formal, Conceituação, Curadoria dos dados, Escrita – primeira redação, Escrita – revisão e edição, Investigação, Metodologia, Obtenção de financiamento, Validação. Medeiros, A.C.L.V.: Curadoria dos dados, Escrita – revisão e edição, Investigação, Metodologia, Validação. Lima, V.M.C.: Curadoria dos dados, Escrita – revisão e edição, Metodologia, Supervisão, Validação, Visualização. Souza, W.V.: Curadoria dos dados, Escrita – revisão e edição, Metodologia, Supervisão, Validação, Visualização. Rego, R.C.F.: Escrita – revisão e edição, Metodologia, Supervisão, Validação, Visualização. Santos, M.O.S.: Administração do projeto, Conceituação, Escrita – revisão e edição, Investigação, Metodologia, Obtenção de financiamento, Supervisão, Validação, Visualização. Gurgel, I.G.D.: Administração do projeto, Conceituação, Escrita – revisão e edição, Investigação, Metodologia, Obtenção de financiamento, Supervisão, Validação, Visualização.
